# Position and Attitude Estimation Method Integrating Visual Odometer and GPS

**DOI:** 10.3390/s20072121

**Published:** 2020-04-09

**Authors:** Yu Yang, Qiang Shen, Jie Li, Zilong Deng, Hanyu Wang, Xiao Gao

**Affiliations:** 1 The School of Mechatronical Engineering, Beijing Institute of Technology, Beijing 100081, China; 2 Beijing Institute of Technology Chongqing Innovation Center, Chongqing 401120, China

**Keywords:** attitude and position estimation, visual odometer, GPS

## Abstract

The monocular visual odometer is widely used in the navigation of robots and vehicles, but it has defects of the unknown scale of the estimated trajectory. In this paper, we presented a position and attitude estimation method, integrating the visual odometer and Global Position System (GPS), where the GPS positioning results were taken as a reference to minimize the trajectory estimation error of visual odometer and derive the attitude of the vehicle. Hardware-in-the-loop simulations were carried out; the experimental results showed that the positioning error of the proposed method was less than 1 m, and the accuracy and robustness of the attitude estimation results were better than those of the state-of-art vision-based attitude estimation methods.

## 1. Introduction

The position and attitude are essential measurements in guidance and control of an Unmanned Aircraft Vehicle (UAV), and they are usually obtained by Inertial Navigation Sensors (INS) [1,2] and being corrected by the GPS (Global Position System) [3,4,5], magnetic sensors [6], and visual odometers [7,8], etc. However, these methods require the initial alignment of INS, leading to the problem that the vehicle needs to stay stationary at the beginning when aligning the INS [9,10]. This problem can be solved by transfer alignment with external attitude measurement equipment [11,12,13], but it would increase the cost and weight on the navigation system.

Monocular cameras are widely used in the visual navigation of UAVs because they have the advantages of being inexpensive and small. For a UAV equipped with a monocular camera, the attitude estimation can be realized without INS, and the methods of estimating the attitude with pure computer vision are studied by many researchers, while a variety of methods have been proposed, such as horizon detection method [14,15] and vanishing points method [16,17,18]. Timotheatos proposed a vehicle attitude estimation method based on horizon detection [19], which detects the horizon line based on a canny edge and a hough detector along with an optimization step performed by a Particle Swarm Optimization (PSO) algorithm. The roll angle of the vehicle is determined from the angle formed by the slope of the horizon, and the pitch angle is computed using the current and the initial horizon positions. This method is further studied by Dumble [20], who extracts the horizon more accurately based on the spatial correlation and removes the straight horizon line assumption. Additionally, Hwangbo proposed an attitude estimation method based on vanishing points [21]. The attitude of the vehicle is estimated based on the assumption that the outlines of buildings are either vertical or parallel to the horizon. However, all these methods take external references to estimate attitude, and they may be inefficient when there are no references or the references are not identical to their assumptions.

In addition to using references to achieve attitude estimation, the attitude of the vehicle can be obtained by another vision-based attitude estimation method, Visual Odometer (VO), also known as the Simultaneous Localization and Mapping (SLAM) method [22]. SLAM has been a hot research topic in the last two decades, and has been developed by many researchers, such as ORB-SLAM series proposed by Raul Mur-Artal [23], SVO series proposed by Christian Forster [24], LSD-SLAM series and DSO series proposed by Jakob Engel [25,26]. However, there are defects of the monocular visual odometer that the scale of the estimated trajectory is unknown [27,28], so it is impossible to obtain the positioning results from pure VO with a monocular camera. Through the combination of a low-cost monocular camera and a low-cost GPS receiver, one could get higher positioning accuracy than the GPS receiver and eliminate the scale uncertainty brought by the monocular camera, and the attitude of the vehicle can be extracted from VO as well.

In this paper, we proposed a position and attitude estimation method, integrating the Visual Odometer and GPS, namely GVO (Visual Odometer integrated with GPS). In GVO, the relative position and attitude were estimated by a monocular visual odometer, and the positioning results obtained by GPS were taken as references to eliminate the scale uncertainty of the pose of camera, where the position and attitude of the vehicle could be obtained simultaneously. The application of this method would not be limited by other factors except the general requirements of GPS and visual odometer. For a long-endurance UAV with a monocular vision-based navigation system, when the attitude is estimated by the INS, it is usually used with GPS to improve the robustness and reduce the error of the system. In the long-distance flight of UAV, when the system suddenly loses power in an emergency and restarts in the air, the alignment of INS is difficult, while the GVO method could still work and help align the INS. Furthermore, with the development of image processing hardware, the proposed algorithm could be implemented on the image processing system of the onboard camera without additional costs. So, as an assistant, backup, and even alternative technology to the INS, the proposed method could greatly improve the robustness and stability of the navigation system with promising advantages.

The structure of this paper is given as follows. In Section 2, the proposed method is described in detail, and the scale factor and rotation matrix between VO and GPS are derived, the optimization of estimation results is studied as well. In Section 3, the proposed method is verified by hardware-in-the-loop simulations, and the experimental results show that the proposed method performs better than traditional visual methods, in which attitude estimation error is less than 2°, and the positioning error is less than 1 m. At last, conclusions are drawn in Section 4.

## 2. The Position and Attitude Estimation Method of GVO

### 2.1. The Principle of GVO

**Figure 1:**
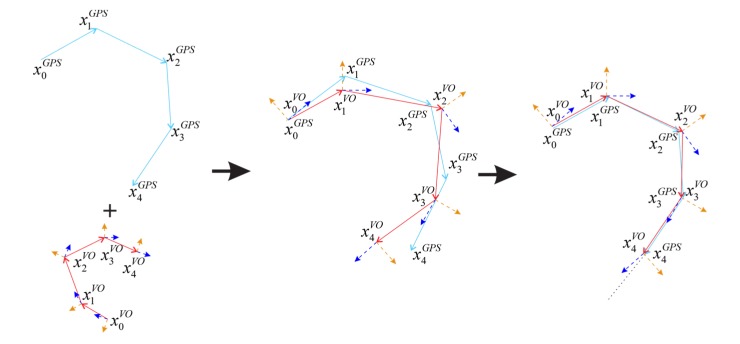
The diagram of integrating Visual Odometer and Global Position System (GPS).

The principle of the proposed method is shown in Figure 1, where the trajectories of the vehicle are simplified from 3D (3-Dimensional) space to 2D (2-Dimensional) space to help understand where the height information in 3D space is omitted here, and the trajectories are represented on a 2D plane. In Figure 1, the light blue lines indicate the trajectories measured by GPS, the red lines represent the motion trajectories estimated by VO, and the broken lines in yellow and dark blue are the attitude estimated by VO. The trajectory of the vehicle is estimated by GPS and Visual Odometer, represented by $x_{n}$ and $x_{n}^{\mathrm{vo}},$ respectively, where *n* represents the index of points. As shown in Figure 1, firstly, the GPS and visual odometer estimate the flight trajectories separately. Secondly, the trajectories estimated by visual odometer and GPS are transformed into the same coordinate system. At last, we assume that the error of GPS measurements $x_{n}$ is unbiased white noise [29] and that there is an accumulative error in the estimated position of VO $x_{n}^{\mathrm{vo}}$. So, the GPS positioning results are taken as measurements of the true position to correct the estimations of VO, and the scale factor between $x_{n}^{\mathrm{vo}}$ and $x_{n}$ is derived. Considering that the pose of the camera obtained by VO contains the attitude information, the attitude of the vehicle can be estimated as well.

### 2.2. The Position and Attitude Estimation

The update frequency of GPS is usually 5–10 Hz. The computational frequency of VO is 25–30 Hz, which is several times of the update frequency of GPS. So, it is necessary to have a hardware alignment or software alignment method to realize the synchronization between the pose $T_{n}$ obtained by VO and $x_{n}$ estimated by GPS.

Let $x_{k}$ be the position measurement of GPS, which has been synchronized with VO at frame *k*, and let $T_{k}$ be the transform matrix estimated by VO between frame *k* − 1 and *k*. For the sake of brevity, 3D coordinates like x=(x,y,z) are automatically transformed into matrix $x={\left[x\;y\;z\;1\right]}^{T}$ when multiplied with transform matrix $T_{k}$. $T_{k}$ is given as follows,

(1) $$T_{k}=\left[\begin{array}{cc} R_{k} & t_{k}\\ 0^{T} & 1 \end{array}\right],$$

where $T_{0}=I\in {\mathbb{R}}^{4\times 4}$, $R_{k}\in \mathrm{SO}\left(3\right)$ is the rotation matrix, SO(3) is the special orthogonal group $\mathrm{SO}\left(3\right)=\{R_{k}\in {\mathbb{R}}^{3\times 3}|R_{k}R_{k}^{T}=I,\det\left(R_{k}\right)=1\}$, $t_{k}\in {\mathbb{R}}^{3}$ is the translation matrix.

The position measurements of GPS are usually indicated by geographic coordinates, longitude $L_{k}$, latitude $B_{k},$ and height $H_{k}$. In order to obtain the attitude angles of pitch and roll, the East-North-Up (ENU) coordinate system is selected to be the intermediate coordinate system to fuse the measurements of GPS and VO, where the transformations between geographic coordinates and ENU frame are given in [30]. After time alignment between GPS and VO, the coordinates of GPS are transformed into the ENU frame $x_{k}^{enu}=\left(x_{k}^{enu},y_{k}^{enu},z_{k}^{enu}\right)$ to integrate with VO.

As the relationship between the initial local frame of VO and ENU frame is certain, the transformation matrix of them is constant. The position and attitude of the camera in the ENU frame can be obtained by using the poses estimated by VO. The transformation between measurements of VO and GPS in the ENU frame can be represented by,

(2) $$R\left(x_{k}^{\mathrm{vo}}-x_{k-1}^{\mathrm{vo}}\right)=K\left(x_{k}^{enu}-x_{k-1}^{enu}\right),x_{k}^{\mathrm{vo}}=\overset{k}{\underset{j=0}{\prod }}T_{j}x_{0}^{\mathrm{vo}},$$

where $x_{k}^{\mathrm{vo}}$ is the position in the local frame of VO at frame *k*, $x_{0}^{\mathrm{vo}}=\left(0\;0\;0\right)$ is the origin in the local frame of VO, and $x_{0}^{enu}=\left(0\;0\;0\right)$ is the origin of GPS measurements in ENU frame. R is the rotation matrix from the local frame of VO to ENU frame, K is a diagonal matrix, and it represents the scale factor from ENU frame to the local frame of VO, in which the three row vectors represent the scale factors in east, north, and up directions, respectively. 

To simplify Equation (2), $t_{k}$ and $d_{k}$ are defined as,

(3) $$t_{k}=x_{k}^{\mathrm{vo}}-x_{k-1}^{\mathrm{vo}}={\left[\begin{array}{ccc} t_{x}^{k} & t_{y}^{k} & t_{z}^{k} \end{array}\right]}^{T},$$

(4) $$d_{k}=x_{k}^{enu}-x_{k-1}^{enu}={\left[\begin{array}{ccc} d_{x}^{k} & d_{y}^{k} & d_{z}^{k} \end{array}\right]}^{T}.$$

Thus, obtaining (5),

$$Rt_{k}=Kd_{k},$$

(5) $$\left[\begin{array}{ccc} r_{11} & r_{12} & r_{13}\\ r_{21} & r_{22} & r_{23}\\ r_{31} & r_{32} & r_{33} \end{array}\right]\left[\begin{array}{c} t_{x}^{k}\\ t_{y}^{k}\\ t_{z}^{k} \end{array}\right]=\left[\begin{array}{ccc} k_{1} & 0 & 0\\ 0 & k_{2} & 0\\ 0 & 0 & k_{3} \end{array}\right]\left[\begin{array}{c} d_{x}^{k}\\ d_{y}^{k}\\ d_{z}^{k} \end{array}\right].$$

For the convenience of calculation, the elements in (5) are moved to the left side as given below,

(6) $$\left[\begin{array}{ccc} r_{11} & r_{12} & r_{13}\\ r_{21} & r_{22} & r_{23}\\ r_{31} & r_{32} & r_{33} \end{array}\begin{array}{ccc} -k_{1} & 0 & 0\\ 0 & -k_{2} & 0\\ 0 & 0 & -k_{3} \end{array}\right]\left[\begin{array}{c} \begin{array}{c} t_{x}^{k}\\ t_{y}^{k}\\ t_{z}^{k} \end{array}\\ \begin{array}{c} d_{x}^{k}\\ d_{y}^{k}\\ d_{z}^{k} \end{array} \end{array}\right]=0,$$

However, there is usually no analytic solution of R and K due to the white noise in GPS measurements and the accumulative error of VO. So, the least square method is used to solve this equation. To obtain R and K, define the solution vector m as follows,

(7) $$m={\left[r_{11}\;r_{12}\;r_{13}\;r_{21}\;r_{22}\;r_{23}\;r_{31}\;r_{32}\;r_{33}-k_{1}-k_{2}-k_{3}\right]}^{T}.$$

When measurements of GPS and VO from frame 1 to frame *k* are introduced into (6), it can be rewritten as,

(8) $$\left[\begin{array}{ccccccc} t_{T}^{k} & & & & d_{k}^{x} & & \\ & t_{T}^{k} & & & & d_{k}^{y} & \\ & & t_{T}^{k} & & & & d_{k}^{z}\\ \vdots & \vdots & \vdots & & \vdots & \vdots & \vdots \\ t_{T}^{1} & & & & d_{1}^{x} & & \\ & t_{T}^{1} & & & & d_{1}^{y} & \\ & & t_{T}^{1} & & & & d_{1}^{z} \end{array}\right]m=0.$$

As shown in (6), each pair of positioning results of GPS and VO gives three formulas, while there are 12 unknown variables. So, this over-determined equation could be solved by using more than four pairs of positioning results of GPS and VO, and the solution vector m can be obtained after the fourth frame of the input image sequence. Then, the estimated matrices $\tilde{R}$ and $\tilde{K}$ are given as follows,

(9) $$\left[\begin{array}{cc} \tilde{R} & -\tilde{K} \end{array}\right]\left[\begin{array}{c} \begin{array}{c} t_{x}^{k}\\ t_{y}^{k}\\ t_{z}^{k} \end{array}\\ \begin{array}{c} d_{x}^{k}\\ d_{y}^{k}\\ d_{z}^{k} \end{array} \end{array}\right]=0,$$

where $\tilde{R}$ is the estimated nonorthogonal matrix that approximates the orthogonal matrix R. Since $\;\tilde{R}$ does not always meet orthogonality condition, a further process of $\tilde{R}$ needs to be done, and (9) is divided by $\left|\tilde{R}\right|$, as shown in (10).

(10) $$\hat{R}=\tilde{R}/\left|\tilde{R}\right|,K=\tilde{K}/\left|\tilde{R}\right|.$$

Here, the scale factor K is obtained in this equation. But $\hat{R}$ may still not be orthogonal, and we have to find an orthogonal matrix, which is the most approximate to $\hat{R}$, where the Singular Value Decomposition (SVD) method is used to solve this problem, and it is given as follows,

(11) $$\hat{R}=U\Sigma V^{T},\;\;R=UV^{T},$$

where U, V are the orthogonal matrices, Σ is a diagonal matrix with positive elements.

In this way, R is calculated by using more than four pairs of positioning results of GPS and VO, where the selected points must not be on a straight line. Because the linear relationship between the points would produce infinite solutions, the selection of the points can be conducted by using the positioning results of GPS; as long as the they are not on a straight line, they can be used to calculate R. When there are more data involved in the calculation after a period of flight, the results can be more accurate. This is one of the limitations of the proposed method that the positioning results of GPS and VO used to calculate R and K must not be on a straight line. Another limitation is the defect of VO that the ground must appear in the view of the camera, and if there are only ocean and sky, it will lead to large errors in the estimations of VO.

Then, the orientation $R_{k}$ in the pose $T_{k}$ of VO can be used to derive the attitude of the vehicle $a_{k}$ in the ENU frame, which is given by

(12) $$a_{k}=RR_{k}.$$

Through the equations above, the rotation matrix and scale factor between GPS and VO are obtained, and the attitude of the vehicle is derived as well.

### 2.3. The Optimization of GVO

However, the rotation matrix and scale factor between GPS and VO derived in the last section are not optimal solutions because there are accumulative errors in the poses obtained by VO, which would lead to a large error in the estimated rotation matrix and scale factor. In order to reduce the error of estimated position and attitude as much as possible, the optimization process has to be done. 

According to (5), the position measurements of VO in the ENU frame are given as follows,

(13) $$x_{k}^{\mathrm{ve}}-x_{k-1}^{\mathrm{ve}}=K^{-1}R\left(x_{k}^{\mathrm{vo}}-x_{k-1}^{\mathrm{vo}}\right),$$

where $x_{k}^{\mathrm{ve}}\in {\mathbb{R}}^{3}$ is the position estimated by VO, which has been transformed to the ENU frame.

After the transformation in (13), the optimization process is carried out, and the proposed optimization algorithm is given in Algorithm 1.
**Algorithm 1** Optimization of GVO**Require:** Positioning results of GPS $x_{k}^{enu}$ and poses of camera $T_{k}$.1: Optimize R and K by minimizing error between GPS measurements $x_{k}^{enu}$ and the positions estimated by VO $x_{k}^{\mathrm{ve}}$ with initial values obtained by (10) and (11).2: Optimize the poses $T_{k}$ of camera by minimizing error between GPS measurements $x_{k}^{enu}$ and the positions estimated by VO $x_{k}^{\mathrm{ve}}$.3: Use BA (Bundle Adjustment) to optimize the camera poses $T_{k}$ again by minimizing the image reprojection error between the matched 3D points in ENU frame and their keypoints in the image.4: **if** The optimization converges **then**5: **return** The optimized R**,**
K
**and**
$T_{k}$.6: **else**7: Repeat step1 to step 68: **end if**


There are three major steps of the optimization process. Firstly, taking R and K obtained by (10) and (11) as the initials to help accelerate the convergence of optimization. They are optimized by minimizing errors between GPS measurements and the positions estimated by VO, which is given by,

(14) $$\left\{R,K^{-1}\right\}=\underset{R,K^{-1}}{\mathrm{argmin}}\underset{k}{\sum }\|x_{k}^{\mathrm{enu}}-x_{k}^{\mathrm{ve}}{\|}_{2}^{2}=\underset{R,K^{-1}}{\mathrm{argmin}}\underset{k}{\sum }\|x_{k}^{\mathrm{enu}}-(x_{k-1}^{\mathrm{ve}}+K^{-1}R{\left(x_{k}^{\mathrm{vo}}-x_{k-1}^{\mathrm{vo}})\right\|}_{2}^{2}.$$

Let $T_{k}^{\mathrm{ve}}$ be the poses of VO, which have been transformed to the ENU frame,

(15) $$T_{k}^{\mathrm{ve}}=\left[\begin{array}{cc} R_{k}^{\mathrm{ve}} & t_{k}^{\mathrm{ve}}\\ 0^{T} & 1 \end{array}\right]=K^{-1}RT_{k}.$$

After the optimization of R and K, the poses of the camera $T_{k}^{\mathrm{ve}}$ at each frame are optimized by minimizing the errors between the GPS positioning results and the poses estimated by VO, which is represented by,

(16) $$T_{k,0}^{\mathrm{ve}}=\overset{k}{\underset{j=0}{\prod }}T_{j}^{\mathrm{ve}},\;x_{k}^{\mathrm{ve}}=T_{k,0}^{\mathrm{ve}}x_{0}^{\mathrm{ve}},$$

(17) $$\left\{T_{k,0}^{\mathrm{ve}}\right\}=\underset{T_{k,0}^{\mathrm{ve}}}{\mathrm{argmin}}\sum_{k}\|x_{k}^{\mathrm{enu}}-T_{k,0}^{\mathrm{ve}}x_{0}^{\mathrm{ve}}{\|}_{2}^{2}.$$

At last, we use BA (Bundle Adjustment) to optimize the camera poses by minimizing the image reprojection error between the matched 3D points in ENU frame and their key points in the image, where the method is referenced in [31]. The position of the ground mark $x_{\mathrm{mark}}^{\mathrm{ve}}$ in the ENU frame is given by

(18) $$x_{\mathrm{mark}}^{\mathrm{ve}}=x_{0}^{\mathrm{ve}}+K^{-1}R\left(x_{\mathrm{mark}}^{\mathrm{vo}}-x_{0}^{\mathrm{vo}}\right),$$

where $x_{\mathrm{mark}}^{\mathrm{vo}}$ is the position of ground mark in the local frame of VO.

After the optimization process above, the system would judge whether the optimization result converges or not. If it converges, the optimization result will be output. Otherwise, perform the optimization process again until it converges.

## 3. Hardware-in-the-Loop Simulation

### 3.1. The Experimental Environment

**Figure 2:**
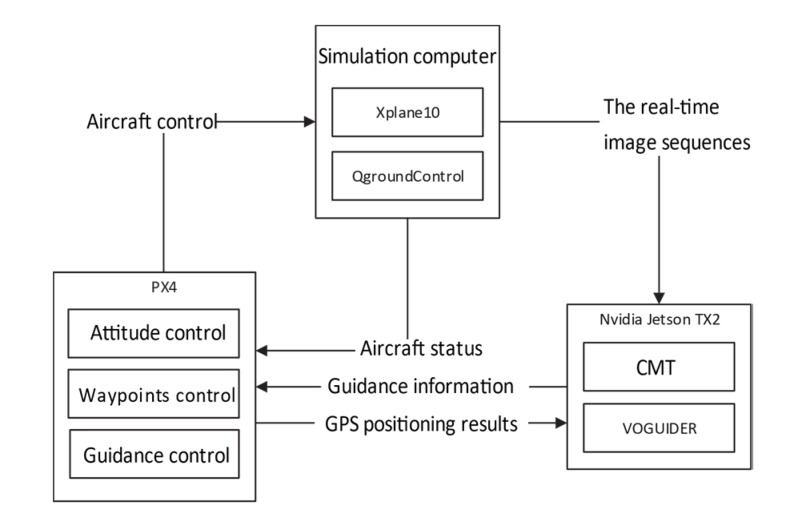
Block diagram of the simulation system.

Hardware-in-the-loop simulations were conducted to verify the proposed method, and the block diagram of the simulation system is shown in Figure 2.

As shown in Figure 2, the simulation system consisted of three parts—the simulation computer, PX4 embedded flight controller, and Nvidia Jetson TX2 embedded image processing board. The computer was used to simulate the visual environment, the PX4-embedded flight controller was used to control the attitude of aircraft, and the Nvidia Jetson TX2-embedded image processing board was used to run the GVO method. The CPU of the computer was Intel i7-7700K 4.20 GHz, the GPU of the computer was Nvidia GTX 1080 8 G, and the RAM of the computer was 32 GB. The integral proportional guidance law was added to the PX4-embedded flight controller with existing codes to help guidance control, where the codes could be downloaded on the website [32]. The size and weight of the TX2 module were 50 × 87 mm and 85 g, respectively. The power of TX2 when running the algorithm was 20 W and the cost of it was $399.

**Figure 3:**
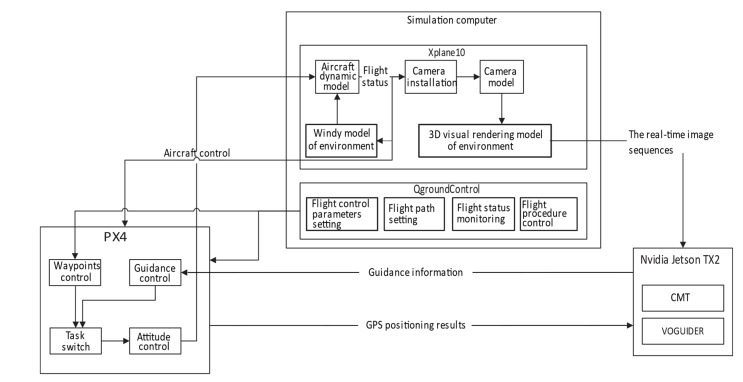
Block diagram of the working process of the simulation system.

The block diagram of the working process of the simulation system is shown in Figure 3. First, the 3D environment was rendered by Xplane10 on the computer. The real-time image sequences were captured during flight by setting the installation and intrinsics of the camera. Then, the image sequences would be transmitted to the NVIDIA image processing board to run the proposed GVO method and get the estimated position and attitude of UAV, where the GPS positioning results were obtained from the PX4 flight controller. The estimated position and attitude were fed back to PX4 so that it could realize attitude control using the preset waypoints of the Qground Control software on the computer and give the attitude control message to Xplane10. When Xplane10 received the attitude control message from PX4, it changed the flight status of the aircraft, while the dynamic model of the aircraft and the windy model of the environment were taken into account. In addition, the flight status of the UAV would be fed back to PX4 to form a closed loop, which would assist PX4 to control the attitude of the UAV during flight. The update frequency of the PX4 controller, the image output frequency of XPlane 10, and the image sampling frequency of NVIDIA Jeston were all 50 Hz.

### 3.2. Positioning Results of GVO

To verify the positioning accuracy of GVO in different conditions, multiple heights were employed to conduct simulations, and the simulation conditions are shown in Table 1.

The flying speed in Table 1 is the cruising speed of MyTwinDream, a fixed-wing Unmanned Aircraft Vehicle (UAV), manufactured by FPVmodel in Xiamen China. Due to the disturbance of wind, the speed of UAV was not fixed during flight. The camera parameters here were the parameters of a commonly used onboard camera.

**Figure 4:**
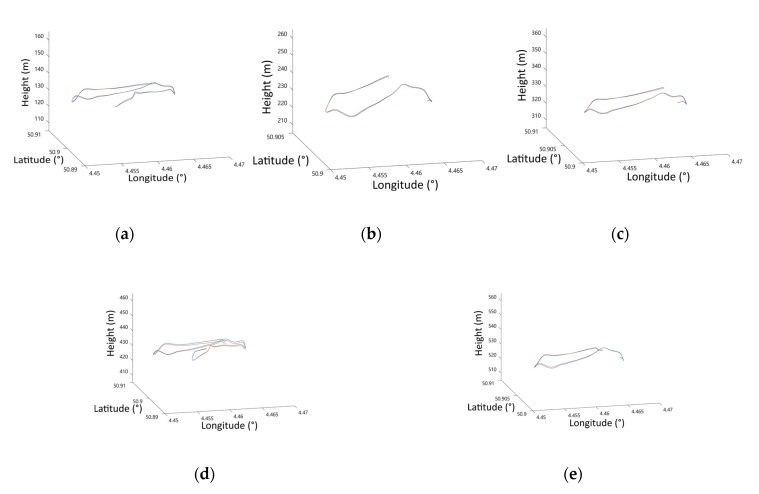
The trajectory estimation results. (**a**) 100 m height. (**b**) 200 m height. (**c**) 300 m height. (**d**) 400 m height. (**e**) 500 m height.

The positioning results of GVO and GPS are shown in Figure 4, where the blue lines are estimated by GVO, and the red lines are measurements of GPS with a standard variance of 5 m.

**Figure 5:**
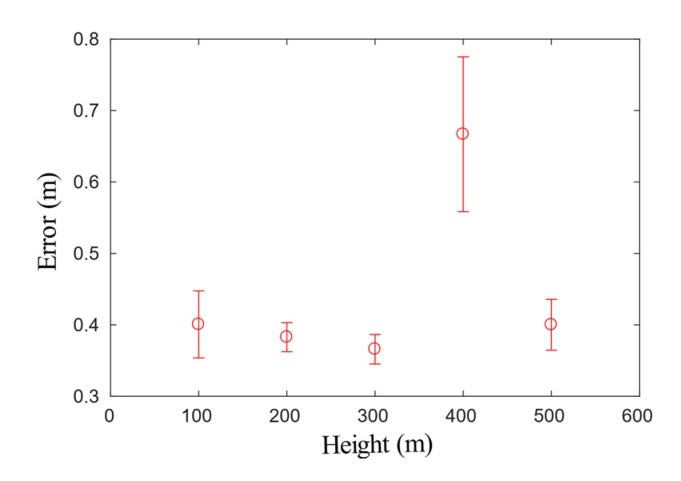
The bar diagram of positioning error.

As we could see from Figure 4, the trajectories estimated by GVO basically overlapped with the original GPS measurements. The statistics of the positioning errors of GVO are given in Table 2, and the corresponding bar diagram is shown in Figure 5.

As we could see from Table 2 and Figure 5, the mean deviation of positioning results was less than 1 m, and the standard variance of GVO was less than 0.7 m, which was much smaller than that of pure GPS with a standard variance of 5 m. 

**Figure 6:**
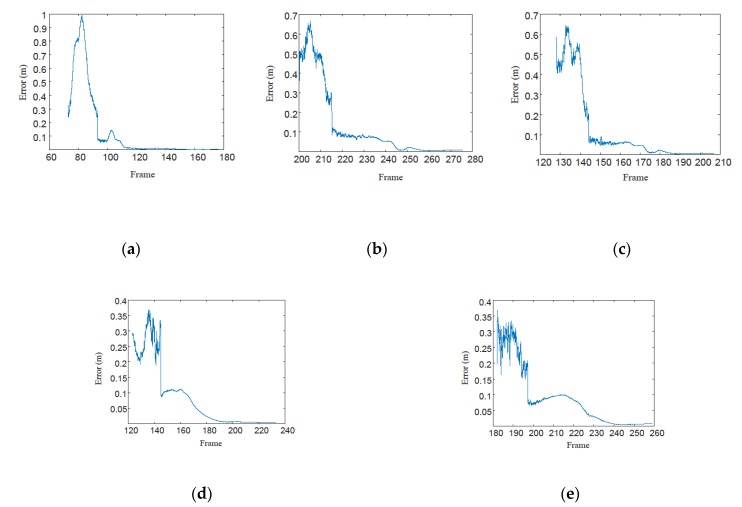
The positioning errors of GVO (Visual Odometer integrated with GPS). (**a**) 100 m height. (**b**) 200 m height. (**c**) 300 m height. (**d**) 400 m height. (**e**) 500 m height.

The convergence property of the proposed method was tested as well. The positioning errors of GVO are shown in Figure 6, and they were convergent in all cases, while the slowest one converged after the 250th frame at the frame rate of 25 frames per second. It showed that the system converged in a short time, which was within 10 s by using the proposed method.

### 3.3. Attitude Estimation Results

To assess the robustness and accuracy of GVO on attitude estimation, this section has compared GVO with two state-of-art monocular visual attitude estimation methods—the horizon detection method, and the vanishing points method.

**Figure 7:**
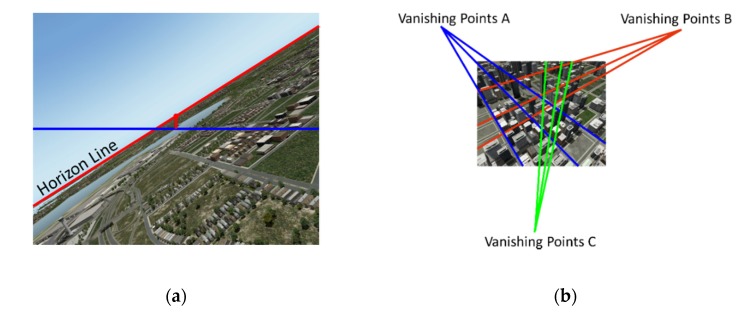
The attitude estimation method. (**a**) The horizon detection method. (**b**) The vanishing points method.

The premise of the horizon detection method for attitude estimation is that the horizon must appear in the view of the camera. This algorithm estimated the roll angle of the camera by detecting the slope of the horizon, and the pitch angle was estimated from a distance between the center of the horizon and the central axis of the camera, which is shown in Figure 7a. The vanishing points method estimated attitude when there were a large number of artificial constructions. The attitude was estimated by the vanishing points of outlines of the buildings when the outlines were supposed to be parallel or vertical to the horizon, as shown in Figure 7b.

Simulations were carried out in multiple environments like the plain, mountain, and city to compare the proposed method with different state-of-art attitude estimation methods, and the experimental conditions are given in Table 3.

**Figure 8:**
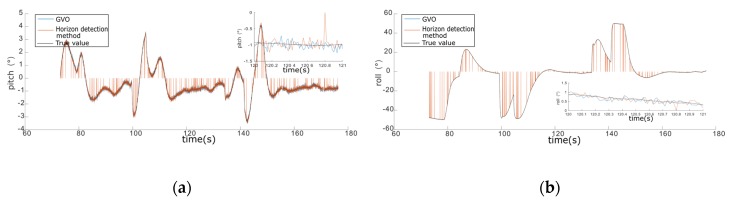

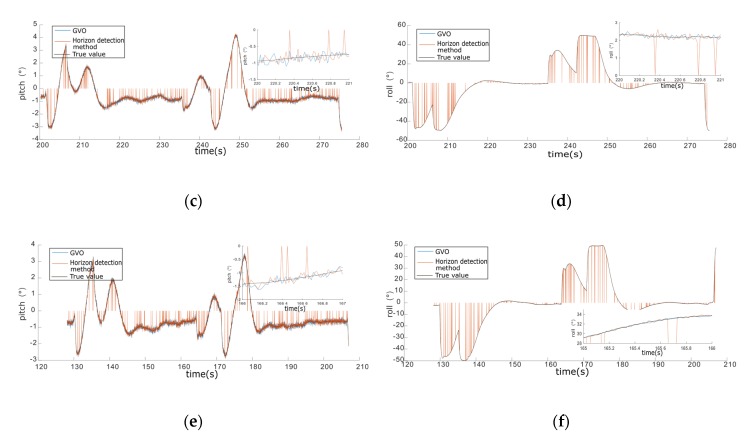
The simulation results of GVO and horizon detection method in the plain area. (**a**) Pitch angle of 100 m height. (**b**) The roll angle of 100 m height. (**c**) Pitch angle of 200 m height. (**d**) The roll angle of 200 m height. (**e**) Pitch angle of 300 m height. (**f**) The roll angle of 300 m height.

**Figure 9:**
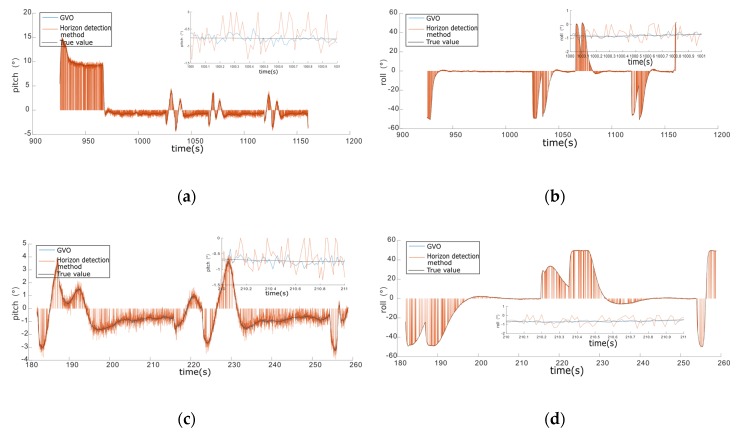

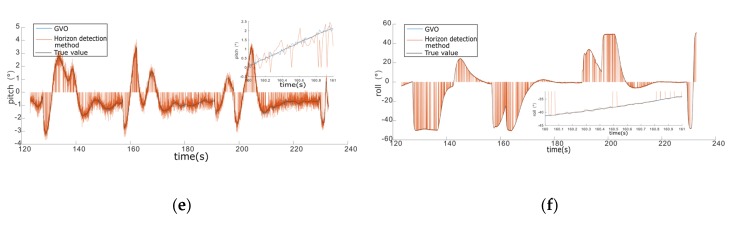
The simulation results of GVO and horizon detection method in the mountainous area. (**a**) Pitch angle of 100 m height. (**b**) The roll angle of 100 m height. (**c**) Pitch angle of 200 m height. (**d**) The roll angle of 200 m height. (**e**) Pitch angle of 300 m height. (**f**) The roll angle of 300 m height.

The horizon detection method was compared with the proposed method in plain and mountain areas, where the experimental results are given in Figure 8 and Figure 9, respectively. As shown in Figure 8, the attitude estimation results of GVO were very close to those of the horizon detection method in the plain area, but the horizon detection method failed occasionally, which was also at the time when the estimated values jumped to zero. The horizon detection method judged whether the detected lines met the length threshold of the horizon to ensure the horizon detection accuracy, and it failed when there were no detected lines that could meet the requirements of the horizon assumption. The GVO method was not affected by the environment, so there were no failures of GVO in the simulations.

The simulation results of GVO and horizon detection method in the mountainous area are shown in Figure 9. As shown in Figure 9, the accuracy and success rate of GVO were obviously higher than that of the horizon detection method. Compared with the plain area, the horizon in the mountainous area was more difficult to be detected. We could see from Figure 9 that the failure frequency of the horizon detection method was much larger than that of the plain area, and the attitude estimation accuracy in the mountainous area was worse. Meanwhile, the GVO still worked properly, which proved that the advantages of GVO were more prominent in the mountainous areas.

The statistics of GVO and horizon detection method in plain and mountain environments are given in Table 4. As shown in Table 4, the accuracy of GVO was a little higher than that of the horizon detection method, but the success rate of GVO was much larger, especially in the mountainous area. As long as VO and GPS could work properly, the success rate of GVO was always 100%, which proved that the proposed method could estimate the attitude efficiently without being influenced by the external environment. So, the GVO method had the advantages of higher accuracy and better robustness than the horizon detection method.

**Figure 10:**
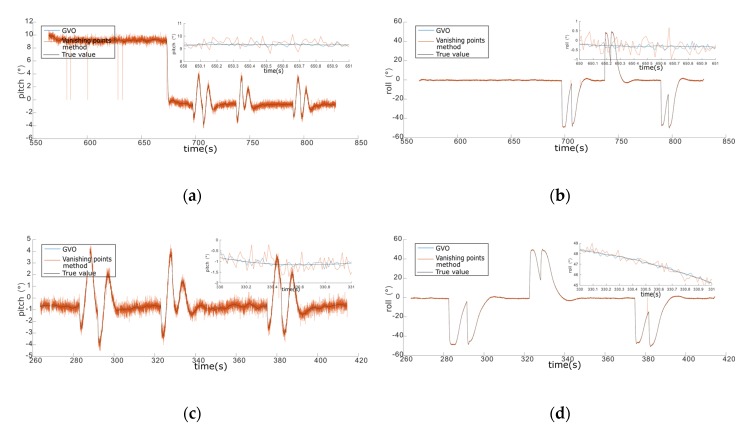
The simulation results of the GVO method and the vanishing points method in the city area. (**a**) Pitch angle of 100 m height. (**b**) The roll angle of 100 m height. (**c**) Pitch angle of 200 m height. (**d**) The roll angle of 200 m height.

As shown in Figure 10, simulations in the city were conducted to compare GVO with the vanishing points method, and the statistics are given in Table 5. As we could see from Figure 10 and Table 5, the success rate of the vanishing points method decreased sharply with the increase of height, while the success rate of the GVO method was still 100%. When the height increased, the outlines of the buildings got shorter, and the detection accuracy of the vanishing points method would be reduced when the lengths of the lines were too short to meet the detection threshold. The GVO was not affected by the variation of height, and it had higher attitude estimation accuracy with an average error of 1.2°, while the average error of the vanishing point method was 2.9°.

Overall, GVO showed better performance of accuracy and robustness in attitude estimation than the state-of-art methods. From the simulations above, the success rate of GVO in attitude estimation was always 100% in spite of the variation of environment and height. In comparison, the average success rates of the horizon detection method and vanishing points method were 91.3% and 87.5%, respectively. The attitude estimation error of GVO ranged from 1.1° to 1.3°, while the error of horizon detection method ranged from 2.0° to 2.1° in plain area and 2.2° to 2.5° in the mountainous area. The average error of vanishing points method ranged from 2.7° to 3.1° in the city. 

## 4. Conclusions

In this paper, an algorithm, integrating the Visual Odometer and GPS, was proposed to estimate the position and attitude of UAV. The integration method of VO and GPS was described, and the estimations of the proposed method were optimized as well. Hardware-in-the-loop simulations were conducted; the TX2 image processing board that we used was released in 2016, and there are alternative image processing chips with smaller sizes, such as Huawei Hi3559A with the size of 38 × 38 mm, manufactured by Hisilicon in Shanghai China, which is more suitable to be applied on UAV. As such chips enable this method to be applied in engineering. The experimental results showed that the mean deviation of positioning results of GVO was less than 1 m, and the standard variance was less than 0.7 m. Simulations compared with two state-of-art visual attitude estimation methods were carried out to verify the proposed method on attitude estimation, where the results showed that the GVO method performed better in both accuracy and robustness. 

## Figures and Tables

**Table 1:** The simulation conditions.

Number	Flying Height	Flying Speed	Field Angle of Camera	Resolution of Camera
1	100 m	30 m/s	45° × 45°	1024 × 1024
2	200 m	30 m/s	45° × 45°	1024 × 1024
3	300 m	30 m/s	45° × 45°	1024 × 1024
4	400 m	30 m/s	45° × 45°	1024 × 1024
5	500 m	30 m/s	45° × 45°	1024 × 1024

**Table 2:** The aircraft positioning error.

Height/m	Mean Deviation/m	Deviation Variance/m^2^
100	0.41	0.13
200	0.37	0.08
300	0.36	0.08
400	0.67	0.17
500	0.41	0.11

**Table 3:** Experimental conditions.

Number	Area	Height	Algorithms to Compare
1	Plain	100	Horizon detection method
2	Plain	200	Horizon detection method
3	Plain	300	Horizon detection method
4	Mountain	100	Horizon detection method
5	Mountain	200	Horizon detection method
6	Mountain	300	Horizon detection method
7	City	100	Vanishing points method
8	City	200	Vanishing points method

**Table 4:** Comparison between GVO (Visual Odometer integrated with GPS) and horizon detection method.

Algorithm	Area	Height/m	Success Rate	Average Error/°
GVO	Plain	100	100%	1.2
Horizon detection	Plain	100	97%	2.0
GVO	Plain	200	100%	1.1
Horizon detection	Plain	200	98%	2.1
GVO	Plain	300	100%	1.3
Horizon detection	Plain	300	97%	2.0
GVO	Mountain	100	100%	1.2
Horizon detection	Mountain	100	83%	2.5
GVO	Mountain	200	100%	1.1
Horizon detection	Mountain	200	85%	2.3
GVO	Mountain	300	100%	1.1
Horizon detection	Mountain	300	91%	2.2

**Table 5:** Comparison between GVO and vanishing points method.

Algorithm	Height	Success Rate	Average Error/°
GVO	100	100%	1.3
Vanishing points	100	93%	3.1
GVO	200	100%	1.1
Vanishing points	200	82%	2.7
